# Nutrition care practices of primary care providers for weight management in multidisciplinary primary care settings in Ontario, Canada - a qualitative study

**DOI:** 10.1186/s12875-018-0760-3

**Published:** 2018-05-22

**Authors:** Stephanie Aboueid, Ivy Bourgeault, Isabelle Giroux

**Affiliations:** 1 School of Public Health and Health Systems, Faculty of Applied Health Sciences, University of Waterloo, 200 University Avenue West, Waterloo, ON N2L 3G1 Canada; 2 Department of Health Systems, Telfer School of Management, University of Ottawa, 55 Laurier Avenue East, Ottawa, ON K1N6N5 Canada; 3 School of Nutrition Sciences, Faculty of Health Sciences, University of Ottawa, 25 University Private, Ottawa, ON K1N6N5 Canada

**Keywords:** Obesity, Primary health care, Qualitative research, Family medicine, Family physicians, Nurse practitioners, Weight management, Nutrition

## Abstract

**Background:**

Despite the recommended guidelines on addressing diet for the management and prevention of obesity in primary care, the literature highlights that their implementation has been suboptimal. In this paper, we provide an in-depth understanding of current nutrition-related weight management practices of primary care providers (PCPs) working in relatively new multidisciplinary health care settings in Ontario.

**Methods:**

Three types of multidisciplinary primary care settings were included (2 Family Health Teams, 3 Community Health Centres and 1 Nurse Practitioner-Led Clinic). Participants (*n* = 20) included in this study were nurse practitioners (*n* = 13) and family physicians (*n* = 7) supporting care for adult patients (18 years or older). In-depth interviews were transcribed, coded and the content was analyzed using an integrated approach.

**Results:**

Our analysis showed that most PCPs used anthropometric measures such as weight for screening patients who would benefit from nutrition counselling with a dietitian. The topic of nutrition was generally brought up either during physical examinations, when patients were diagnosed with a chronic disease, or when blood markers were out of normal range. Participants also mentioned that physical examinations are no longer occurring annually, with most PCPs offering episodic care. All participants reported utilizing dietetic referrals, noting the enablers for providing the referral, which included access to an on-site dietitian. Nonetheless, dietetic referrals were mostly used when patients had an obesity-related co-morbidity. Participants mentioned that healthy eating advice was reinforced during follow-up visits with patients only when there was enough time to do so. Electronic Health Records (EHRs) were utilized to facilitate message reinforcement by PCPs, who perceived EHRs to be helpful for viewing what was discussed in the session with the dietitian.

**Conclusions:**

PCPs mostly used objective measures to screen for patients who would benefit from nutrition counselling rather than diet assessment, which undermines the importance of dietary intake and overemphasizes weight. With physical examinations occurring less frequently, there will be additional missed opportunities for addressing nutrition-related concerns. The presence of a dietitian on site allowed for PCPs to refer patients to nutrition counselling. Having sufficient time during medical visits and EHRs seemed to facilitate message reinforcement by PCPs in follow-up visits with patients.

**Electronic supplementary material:**

The online version of this article (10.1186/s12875-018-0760-3) contains supplementary material, which is available to authorized users.

## Background

Obesity is multifactorial and is strongly associated with many chronic diseases [1, 2]. In 2014, roughly 5.3 million Canadian adults were living with obesity [3]. Obesity has been classified as a complex, chronic, relapsing condition, which highlights the importance of its prevention and management [4, 5]. Primary care is the first point of contact for all patients and thus is viewed as the ideal place to address obesity [6]. Given the complexity of obesity, multicomponent interventions (i.e., those addressing diet, exercise, and behavioural therapy) are required in primary care [7–9]. In terms of diet, it has been shown that an individualized medical nutrition therapy program is essential for adherence to nutrition counselling for weight management [10]. Registered dietitians **(**RDs**)** are health professionals whose services have been shown to be effective in managing excess body weight [11–13]. However, many patients who would benefit from nutrition counselling are not aware of this service or do not receive it [14–18]. Moreover, only 37% of Canadian patients report that they have received nutrition counselling in primary care [19].

There are, however, many barriers to accessing RD services due to lack of accessibility and in the Canadian context, cost of care [14–16]. In response to the suboptimal care in health promotion practices - including nutrition care - there has been a shift towards multidisciplinary clinics as part of the primary health care reform in Canada [20]. This transition began in the year 2000 and included an objective to focus on the prevention and management of chronic diseases, including obesity [20]. This shift is seen as an enabler for better utilization of specialized health care professionals, including dietitians [20]. Some of the key multidisciplinary initiatives include Community Health Centres (CHCs), Family Health Teams (FHTs), and Nurse Practitioner-Led Clinics (NPLCs).

While CHCs, FHTs, and NPLCs were established at different times, for different reasons, and follow their own models, they share an important characteristic: the variety of health care professionals who work in the same location. Given the importance of preventing and managing obesity in primary care [6], it is critical to understand how primary care providers working in different types of multidisciplinary clinics deliver nutrition care to adult patients **(**18 years or older**)** with obesity. In light of this, our study was designed to understand nutrition-related weight management practices of family physicians (FPs) and nurse practitioners (NPs) working in multidisciplinary clinics. Although there are many approaches to weight management, this study focused on nutrition care in order to provide an in-depth understanding of this practice area while accounting for respondent burden. By capturing the underlying reasons for their practice and contexts in which they work, we can further our knowledge with the aim of improving weight management in primary health care facilities.

## Methods

### Settings and participants

Using a purposive sampling technique, we began by approaching the director of each clinical setting in proximity to the research institution. Once permission was obtained from the clinical director, all primary care providers (PCPs) at each site were contacted via email. If interested, a consent form outlining the details of the study was provided to the PCP. Out of 15 NPs and 10 FPs who were approached, 13 and 7 accepted, respectively. Our sample included NPs and FPs working in 2 FHTs, 3 CHCs, and 1 NPLC. The higher number of NPs involved in this study was due to the inclusion of the NPLC setting, which is primarily comprised of NPs. The ratio of NPs to FPs is the same for the two other settings (3 NPs and 3 FPs). The recruitment and data collection occurred in Fall 2016 and Winter 2017. Each participant was given a 50-dollar Canadian coffee card as a token of appreciation for taking the time to participate in the study.

To test the interview protocol, a pilot test that included 4 PCPs was conducted. Data from the pilot test were not included in this study because the interview protocol was refined based on participants’ feedback to include more follow-up probes. The first inclusion criterion was: NPs and FPs who provide health care to adult patients. This is important as questions that were asked during the interview pertain to excess weight in adult patients. The second inclusion criterion was: NPs and FPs who had been working at the site for at least 6 months. This ensured that the health care professional had been working at the site for a considerable number of hours in order to accurately convey their practices at the clinical setting.

### Data collection

This qualitative study was based on in-depth individual semi-structured interviews. In order to allow for comparability between participants while allowing the interviewer to explore subjects of interest, the combination approach recommended by Patton was used [21]. As such, the interview began by using a standardized open-ended approach and ended with the interviewer being free to explore any subjects of interest during the latter parts of the interview [21]. The main topic areas covered in the interview guide were: 1) the screening tools used for determining which patients would benefit from nutrition counselling, 2) approaching the topic of nutrition and providing initial advice, 3) providing dietetic referrals, and 4) understanding message reinforcement practices (i.e., reinforcing healthy eating advice over time). Questions on barriers and enablers for approaching the topic of nutrition counselling for weight management and providing a dietetic referral were also asked. The interview guide was included as an additional file (Additional file 1).

The interviewer who conducted all the interviews (SA) was a dietitian and graduate student trained in qualitative research methods. A research assistant (RA) was also present during the interview and took detailed notes of the conversation as well as other context-information. The research assistant was an undergraduate student who wanted to gain experience in research. Her presence did not affect data collection as the research study mostly included notes taken by the  interviewer (SA). Both notes were compared after the interviews for training purposes but RA notes were not used for the analysis. Interviews were recorded with each participant’s written informed consent. Interview length ranged from 30 to 50 min. In the consent form, each participant had the option of providing a member check once data analysis was complete. Seven out of 20 participants were initially interested in member checking.

### Data analysis

The interviews were transcribed verbatim and managed using NVivo software (QSR International Pty Ltd. Version 11). Investigator triangulation was used where researchers used various angles to interpret the data [22]. Two researchers (SA and IG) analyzed the data independently and discrepancies were resolved by including the perspective of a third researcher (IB). Emerging themes were discussed with the research team to minimize bias and maintain reflexivity [23]. An integrated approach was used where the literature review informed both the interview questions and deductive codes, while inductive codes emerged from the data collected. Examples of deductive codes under the theme “barriers for approaching the topic of nutrition” included “lack of time; lack of knowledge”. Nevertheless**,** to avoid forcing data under predetermined codes, inductive codes were also generated based on the interviewees’ responses.

Interviews from all clinical sites were coded together to achieve the main goal of understanding nutrition care practices in multidisciplinary settings, while maximizing diversity. The inductive analysis process consisted of starting with a line-by-line coding approach. Examples of line-by-line codes included “Body Mass Index and body weight”. Once all descriptive codes were identified, similar codes were grouped under 4 main categories (i.e., screening, approaching the topic of nutrition, dietetic referrals, and reinforcing the healthy eating advice). For instance, the codes “Body Mass Index and body weight” were grouped under the category “screening”. Codes changed and developed as more interviews were conducted and as field experience was gained. Constant comparative analysis was used to group major themes, which were then refined in an iterative process. Major themes and interpretations are provided with interview quotes along with the participant’s profession and place of work. Out of the seven participants who were initially interested in a member check (i.e., verifying the way in which the data were analyzed), four participants completed the member check. There were no revisions that needed to be made.

## Results

A total of 20 individual interviews were conducted with primary care providers working in different types of multidisciplinary primary care settings. Table 1 describes the sample in terms of characteristics and demographics.

**Table 1:** Participant characteristics and demographics

Profession	n
Family physicians	7
Nurse Practitioners	13
Experience in profession (years)	n
≤ 5	6
6–15	9
15–25	2
≥ 25	3
Self-identified gender	n
Female	16
Male	4
Experience in organization (years)	n
≤ 1	2
2–5	8
6–10	7
≥ 11	3

### Screening

Participants were asked about how they screened for patients who would benefit from nutrition counselling. Although a particular screening tool was not used, the following measures, markers, or questions were used to screen patients: body mass index (BMI) or body weight, general questions on food intake, blood work tests results, the presence of chronic disease, and waist circumference.

“Dietary intake is always done in the context of weight. If the patient is skinny and has a poor diet we won’t talk about it. If the person is normal weight, we will not bring it up.” FHT, FP – participant 10

Most PCPs did not use an evidence-based screening tool to screen for patients who would benefit from nutrition counselling. Rather, participants mentioned asking general questions to assess diet. As one participant stated:

“I don’t use any tools because I am always dealing with multiple issues so it’s more of a time thing. In the general assessment I ask: ‘do you eat healthy?’ and then they say yes so then I challenge them and ask ‘what is healthy?’ and then they would say ‘I eat Wendy’s instead of McDonald’s and often it’s not really what I am looking for.” FHT, FP – participant 15

### Approaching the topic of nutrition

Participants enumerated many instances in which the topic of nutrition would be brought up as well as how they provided initial advice. The topic of nutrition in terms of weight management was discussed during: physical examinations, when the patient was diagnosed with a chronic disease such as diabetes or hypertension, when blood work indicated hypercholesterolemia or hyperglycemia, if BMI was over 30.0 kg/m^2^, when patients brought it up, during menopause, every medical encounter, and during pregnancy.

As mentioned by most participants, the topic of nutrition was mostly brought up during physical examinations. Participants, however, also reported that these examinations were no longer conducted annually. Episodic care is now favoured where patients receive physicals every two to three years.

“The topic is usually brought up either if they’re in for an annual physical or a problem affected by obesity like diabetes, hypertension, or sleep apnea. Physicals are now every 2 or 3 years and I am going to stop them. Most family doctors are not doing them anymore and are moving towards episodic care.” FHT, FP – participant 14

Most participants felt that nutrition was discussed more in terms of management of chronic diseases rather than prevention. Many reasons for this occurrence were identified including fear of offending patients. However, they believed that a patient’s chronic disease diagnosis justified a discussion regarding nutrition.

“Quite commonly if we’re doing chronic disease management – diabetes, hypertension, lipid control – those types of things come up very early in the conversation. Some people associate weight discussion as a negative thing, instead of something that carries them forward into a positive role for their health management.” NPLC, NP – participant 5

Blood work test results indicating elevated blood cholesterol and/or glucose concentrations, high blood pressure, and an elevated BMI were also indicators that prompted PCPs to bring up the topic of nutrition.

“It’s usually during physicals if I see that their BMI is above 30 or if their A1C is in the diabetic or pre-diabetic range or if they have dyslipidemia.” CHC, FP – participant 21

The patients also seemed to play an important role in the discussion of nutrition as some were reported to be interested in learning more about healthy diets. This was especially the case when they were diagnosed with a chronic disease.

“Obviously sometimes patients will bring it up and say that weight is an issue and will say that they have problems with good diets or what they should eat. It happens quite frequently when people have early pre-diabetes or diabetes. When they get diagnosed more often they are overweight and the first question they ask is what should I be eating.” FHT, FP – participant 10

A patient being diagnosed with a chronic disease was the most mentioned enabler for approaching the topic of nutrition, whereas lack of time was the most important barrier. The enablers and barriers for approaching the topic of nutrition and supporting quotes are provided in Table 2.

**Table 2:** Key enablers and barriers for approaching the topic of nutrition

Key enablers	Examples of quotes from primary care providers
Chronic disease diagnosis	“Sadly, when people have a chronic illness, it is much easier to talk about nutrition. I find the blood pressure helps, if there is an increase; I talk about weight, diet, and exercise.” CHC, NP – participant 20
Patients showing interest	“I would say a lot of times patients actually bring up the topic if I am talking to them about their cholesterol, they will ask what they can do that is not medication. If I am talking about diabetes or cholesterol, I let them know about the non-medical management, which is obviously the preferred route because there are no side effects as opposed to medications that have side effects.” CHC, FP– participant 17
Dietitian on site	“If somebody is coming in and they are here for a prescription renewal, it’s hard to focus time but because I do have the option to refer them to the dietitian, it’s a huge help.” NPLC, NP – participant 1
Out of normal range blood test markers	“If cholesterol is elevated, glucose is elevated, fatty liver based on lab results, regardless of the age of the person. If none of these issues are there, it is possible that I would not bring it up.” NPLC, FP – participant 9
Having access to handouts	“I like handouts because sometimes I know they are not necessarily listening and taking in the information as I am giving them. Let’s say their blood pressure is out of whack and they don’t want to come back to see the dietitian, I will print out the handout.” NPLC, NP – participant 1
Trusting relationship with the patient	“A trusting relationship between the health professional and the patient is number one.” CHC, NP – participant 19
The whole family has obesity	“When more than one family member has obesity, it is easier to bring up the topic of nutrition.” FHT, NP – participant 12
Key barriers	Examples of quotes from primary care providers
Lack of time	“Time consuming and we only have 15-min appointments so sometimes there is no time.” FHT, NP - participant 14
Patients not open to discussing it	“There are many clients who don’t want to hear about it. They’re unstably housed, they’re in abusive relationships, they have a lot of priorities and talking about nutrition and weight management is not among them.” CHC, NP – participant 18
Lack of rapport with the client	“Sometimes it’s the rapport. Some patients don’t care to interact.” NPLC, NP – participant 1
Competing demands	“They just have so many complex issues, mostly psychosocial issues that are predominant in their daily lives that nutrition is not something I can bring up.” CHC, NP – participant 19
Patient perceiving they already know what they need to change	“Some patients will say: yeah yeah, I’ve been told all this before, I know what to do, I just need to do it.” NPLC, NP – Participant 3
Low comfort level of provider to address nutrition	“Some providers may not be as comfortable because they think it is a sensitive topic but really if you just open a dialogue about it often times it will be OK to talk about it (nutrition).” NPLC, NP – Participant 2
Patients not understanding the implications of excess body weight	“I think that some patients may not understand some of the health consequences that could occur due to excess weight and unhealthy lifestyle behaviours” NPLC, NP – Participant 7

### Dietetic referrals

#### Approaching the dietetic referral

PCPs used many different approaches as well as a combination of approaches when suggesting a dietetic referral. These approaches included: assessing patients’ level of readiness to change, proposing the referral while explaining the importance, explaining what a RD does, and providing general information on how their weight is affecting their health.

“Explaining in more detail why the referral is being made and how important it is in disease management and prevention and explain that lifestyle changes can really affect a lot of organs and multiple diseases.” FHT, FP – participant 15

“If I think that their diabetes is out of control because of their weight I will explain that this is getting out of control because no matter what we give you {medication}, you’re intake is going to super exceed that. … We talk about the complications. … So I think it’s general counselling on what the weight is doing to their health.” FHT, FP – participant 10

In particular, one participant elaborated on a new approach of not giving an option to the patient as it increased the likelihood of initiating nutrition counselling with a dietitian.

“I actually changed my technique when it comes to referring to the RD. If I give them the option, more often they are going to decline. I’ve actually changed how I bring it up. I say: I would like you to see the RD, I think she would be able to give you some good advice, I think she can give a good assessment of how your diet could be affecting your weight, cholesterol, blood pressure.” NPLC, NP – participant 1

When asked if they believed their patients were convinced of the importance of dietetic referrals, some felt that most patients did not understand the importance of the referral. Some PCPs felt that the importance of making lifestyle changes is understood over time.

“I feel that they understand the importance over time. So if they are coming in for back pain, and then another time for knee pain, or trouble sleeping. When they start complaining about different things then I can kind of rule out everything else and come back on the topic of nutrition and their weight. Sometimes the light goes off and they say: oh really, I didn’t realize it was that important, I didn’t realize how many aspects of my mental wellness or physical wellness that are contributed to that (nutrition and diet).” CHC, NP – participant 19

#### Instances in which a dietetic referral was provided

Most referrals seemed to be made when the patient was diagnosed with an obesity-related chronic disease rather than obesity alone. Table 3 outlines the instances in which dietetic referrals were made.

**Table 3:** Instances in which a dietetic referral is provided by primary care providers

Themes	Examples of quotes from primary care providers
Patient asking for the dietetic referral	“If they are asking about weight loss I would then tell them that we have a RD for some counselling regarding weight.” CHC, NP – participant 20
Patient was diagnosed with a chronic disease	“So any new diagnosis I automatically refer to the dietitian. For example, any of the triad of cardiac disease, renal failure disease, diabetes, the lipids; those types of patients I refer.” NPLC, NP – participant 5 “There are only one and a half FTE {Full Time Equivalent} RDs so you want to prioritize people and so secondary and tertiary prevention usually takes a hold. Patients are more motivated when they’re sick and in medicine we don’t value prevention as much.” FHT, FP – participant 10
Patient showing motivation or readiness to change	“Their readiness to change. I don’t go on weight or BMI. It is their readiness. It is the same as smoking; I would never refer for smoking cessation if they are not ready. I bring up the topic but then they have to bring it back up to me and show me that they are ready and committed and want to change. If not the failure rates are close to 100%.” FHT, FP – participant 15
Patient was at risk of developing a chronic disease	“I refer for weight management when it’s related to a medical problem or when they are at risk for a disease to develop” CHC, FP – participant 17
Patient with an elevated BMI	“Everyone that has an elevated BMI, or that come in specifically asking to see the dietitian.” NPLC, NP – participant 4
Patient experiencing pain related to obesity	“For people that are obese, it is something I will bring up, like those pain patients.” NPLC, NP – participant 1

In addition, to get a better understanding of why PCPs made referrals to a RD, we asked about the enablers and barriers for providing such a referral (Table 4). Accessibility to a RD and cost-free service were the main enablers for providing a dietetic referral whereas wait times was mentioned to be a barrier.

**Table 4:** Key enablers and barriers for providing a dietetic referral

Key enablers	Examples of quotes from primary care providers
Increasing access to a dietitian	“It’s very easy here to make a referral since we have a dietitian on site and she is pretty quick to see patients.” FHT, NP – Participant 12 “Access. How quickly they can be seen – not just location access, but also even how quickly they can get on the bandwagon. I find that sometimes if there are long delays – it wears off by the time they get in. Whereas if we have one (dietitian) on site and access is quick, I find it’s received well because of that.” NPLC, NP – participant 5
Increasing patient comfort	“Well it’s on site and it’s not a new environment where they have to meet strangers.” NPLC, NP – participant 6
Cost-free dietitian service at point of care	“They (dietitians) are in the building and it is free for the patient.” FHT, FP – Participant 15
Flexible schedule	“Being on site, free of charge, offered in the evenings so more availability for people working” FHT, FP – participant 14
Having a relationship with the dietitian	“Having a relationship with the dietitian. The more you know their abilities. I know the dietitian here is brilliant and I know that she is located in the clinic so that helps me sell it to the patient rather than saying ‘you might get an appointment in 3 months across the city’.” FHT, NP – participant 11
Key barriers	Examples of quotes from primary care providers
None	“None here but in general it would be cost and transportation, but they are already in to see us, we are ground floor, parking is free, easy access, senior access, wheelchair access. We worked hard to bring down the barriers.” FHT, FP – participant 15
Wait times	“The wait time is 2 weeks so that is sometimes not soon enough because it gives patients time to change their mind but I think it is still good.” CHC, NP – participant 19
Not thinking about making a dietetic referral	“Not thinking of it or making assumptions that the patient would not be interested. But it would still be good to offer it.” NPLC, FP – participant 9
Patient not buying in	“Barriers include patient factors such as patients not buying in.” CHC, FP – participant 21
Patients’ negative perception of the session with the dietitian	“The lecture that people think they’re going to get and the shame element about being overweight.” NPLC, NP – participant 6
Requires the patient to come in again	“It would require another appointment.” NPLC, NP – participant 4
Patients not showing interest	“Some people just aren’t interested; they have hang-ups around weight and dieting and don’t want to talk about it.” CHC, NP – Participant 18
Patients’ lack of time	“Those are typically the working group, that their time is fairly limited with family and work so they want a quick in and out, give me the information and I will do the work.” CHC, NP – Participant 19

### Reinforcing the healthy eating advice

Working in a multidisciplinary clinic and having tools to communicate, such as EHRs, seemed to increase the likelihood of PCPs reinforcing healthy eating advice in follow-up visits with patients. However, this was dependent on PCPs having the time to do so.

“If I have time we talk about what the RD suggested and I’ll see her note in the file and I’ll give a bit of positive reinforcement.”

FHT, FP – participant 14

“And also, the next time I see the person I say, “How did it go with the dietitian?” And if they need a follow up on what was discussed I can look it up in the EHR and I will see that she [dietitian] also assessed their readiness to change and likeliness to make the change, so I think it’s terrific for follow-up.” CHC, NP – participant 18

## Discussion

### Summary of results

Questions on the screening process showed that most PCPs used objective measures, such as BMI and out of normal range blood test markers, for assessing which patients would benefit from further nutrition counselling. Some participants also seemed to ask subjective and non-validated questions to assess diet. The topic of nutrition was mostly brought up during physical examinations, which are infrequent now due to the shift towards episodic care. Similarly, due to competing demands and lack of time, nutrition care was provided when a patient was already diagnosed with a chronic disease or when the patients specifically asked diet-related questions. As for dietetic referral practices, some participants were aware that raising awareness before referring to a dietitian was important for patient adherence to nutrition counselling. However, they did not have adequate time to provide details regarding benefits of changing eating habits before providing a referral. Many enablers for providing a dietetic referral were listed, including the RD being on site and the service being cost-free for patients at point of access. While most participants felt that there were no barriers to providing a dietetic referral, some believed that the wait time to see the RD was an issue. PCPs felt that initiating nutrition counselling is time-sensitive and has to be done when patients are motivated. As such, some PCPs initiated the nutrition counselling themselves due to wait times. PCPs seemed to reinforce healthy eating advice in follow-up visits if time permitted. Reinforcing the dietitian’s message was facilitated with EHRs as PCPs were aware of what was discussed in nutrition counselling sessions.

### Comparison with existing literature

Although current screening practices are aligned with the recommended guidelines on the prevention and management of obesity (e.g., BMI and blood markers) [2], it can raise an issue when thinking of screening for nutrition-related diseases that are not linked to excess weight. Some PCPs mentioned that utilizing screening tools to assess diet might not be feasible due to time constraints. Some were unsure which time-effective and evidence-based questions to ask their patients for assessing their diet. As most PCPs seemed to ask subjective questions, it is important to increase awareness about validated tools such as the Rapid Eating Assessment for Patients (REAP). REAP has been suggested as a tool for health professionals to quickly assess who would benefit from further nutrition counselling [24, 25].

Since most participants mentioned that the topic of nutrition was discussed during physical examinations, the move towards episodic care may decrease health promotion opportunities in primary care. Expecting patients to initiate the topic of nutrition may be an issue if patients are unaware of the consequences of a poor diet and its link to chronic diseases. Moreover, patients have expectations from the primary care provider to ask about weight and to address their concerns by suggesting appropriate measures [26, 27]. Approaching the topic of nutrition only when patients were diagnosed with a chronic disease has been shown in this study and in the literature [28–30]. It also seemed that the discussion was more likely to occur as BMI increased, which is in line with previous findings [27, 31].

In contrast to what is suggested in the literature [32], some participants felt that raising awareness before referring patients to a dietitian had no influence on patient adherence to nutrition counselling. This may be due to the fact that some patients may already know the benefits of managing their weight but are unable to do so given the complexity of the condition and personal circumstances [26]. As highlighted in other studies, participating PCPs were more likely to bring up the topic if they had access to a free on-site dietetic referral option [15, 28]. As such, multidisciplinary clinics seemed to mitigate the barriers highlighted by PCPs working in non-multidisciplinary settings, and enabled PCPs to assist their patients with weight management [27, 33–35]. Wait times and lack of accessibility were also highlighted in previous studies [36, 37]. PCPs not thinking about the referral, patients not buying in, patients’ negative perception of the RD session, and patients not wanting to come in were other barriers mentioned by PCPs. Some of these factors were also highlighted in previous studies [14, 38]. In contrast with previous international findings, none of these barriers included PCPs’ unwillingness to collaborate with other health professionals [39].

After consulting the dietitian and setting out individualized goals, it is recommended that PCPs reinforce these goals in follow-up visits [7, 40]. Given the importance that patients attribute to their PCP’s recommendations, it may help patients adhere to the nutrition care plan [41]. Having a dietitian on site and communication tools such as EHRs facilitated message reinforcement in follow-up visits. Studies have shown that not having a RD on site hinders communication, and in turn, prevents message reinforcement, [36, 37, 42] highlighting the importance of having support from allied health professionals to provide optimal care for obesity management [42, 43]. Although EHRs are perceived to play a role of a messenger [44], this study shows that message reinforcement occurs when PCPs have sufficient time during medical visits.

### Strengths and limitations

This study provides important insights on how PCPs working in relatively new multidisciplinary primary care settings provide nutrition care to adult patients with obesity. Understanding how PCPs address nutrition and elucidating the enablers and barriers for providing nutrition care is crucial to inform the optimization of chronic disease prevention and management. Although the sample size may seem small, it did allow the researchers to reach data saturation indicating that future interviews may not have revealed new information. Moreover, the inclusion of different types of primary care settings allowed for diversity. Findings from this study can be transferred to other similar settings.

This study has some limitations. Although we tried to reach diversity in terms of gender, the sample mostly consisted of females. This may limit applicability to males as research has shown a difference in clinical practice based on gender. As well, this study only included multidisciplinary settings where primary care providers had access to other health professionals. Findings from this study may not be transferred to PCPs working in non-multidisciplinary based clinics.

## Conclusions

Given that PCPs are the gatekeepers of the primary health care system, it is important to address the barriers they have highlighted in this study. Increasing awareness on evidence-based screening tools for diet assessment, providing training on how to discuss nutrition and weight, addressing barriers for providing a dietetic referral by reducing wait times, and encouraging message reinforcement are key for optimizing nutrition care. Importantly, as PCPs move towards providing episodic care, alternative solutions are required to ensure that patients are receiving appropriate preventive care.

## Additional file

Additional file 1: Interview guide. This supplementary file is the interview guide that was used to answer the research question of this manuscript. The interview guide includes questions on how the topic of nutrition is brought up, the enablers and barriers of bringing up the topic of nutrition, the screening tools used, how and when a dietetic referral is provided, how the dietetic referral is approached and how/if healthy eating advice is reinforced. (TXT 2 kb)

## Abbreviations

CHC: Community Health Centre
EHR: Electronic Health Record
FHT: Family Health Team
FP: Family physician
NP: Nurse practitioner
NPLC: Nurse Practitioner-Led Clinic
PCP: Primary care provider
RD: Registered Dietitian
